# Aligning Opportunity and Mission: The Impact of Health Care Contracting on AAAs

**DOI:** 10.1093/geroni/igaf122.2498

**Published:** 2025-12-31

**Authors:** Elizabeth Blair, Suzanne Kunkel, Abbe Lackmeyer

**Affiliations:** USAging, Washington, District of Columbia, United States; Miami University, Oxford, Ohio, United States; Miami University, Oxford, Ohio, United States

## Abstract

Area Agencies on Aging (AAAs) and other community-based aging and disability organizations (CBOs) are increasingly contracting with health care entities to provide integrated care; these contracts can also increase revenue for CBOs and allow them to better serve their communities. As policymakers and other stakeholders increasingly support the formation of these contracting partnerships, it is important to understand their impact on AAAs. This session will describe AAA’s experiences with contracting and its impact on their organizational culture. National data will be shared from the 2023 CBO-Health Care Contracting Survey and a qualitative study on the impact of contracting on AAAs. Thirteen individuals representing 10 AAAs participated in the 2024 qualitative study, which consisted of one in-person focus group and five individual interviews. Participants shared their contracting experiences, how contracting affected their organizational operations and culture, and their change-management strategies. Findings include: the importance of mission alignment in deciding to contract; communicating this decision to staff and leadership; and the impact of health care contracting on staffing, data infrastructure, organizational structure and culture. Findings from the CBO-Health Care Contracting Survey also show how organizational challenges related to contracting—such as staff resistance to change and integration of AAA services into health care system workflow—have changed over time. This session will discuss additional ways that policymakers and others can support AAAs in their contracting efforts, such as providing funding for AAA data infrastructure and capacity development and conducting additional research to identify the elements of a successful contracting partnership.

